# Body Acceptance by Pregnant Women and Their Attitudes toward Pregnancy and Maternity as Predictors of Prenatal Depression

**DOI:** 10.3390/ijerph17249436

**Published:** 2020-12-16

**Authors:** Hanna Przybyła-Basista, Elżbieta Kwiecińska, Michalina Ilska

**Affiliations:** 1 Institute of Psychology, University of Silesia, 40-126 Katowice, Poland; michalina.ilska@us.edu.pl; 2 Psychological and Pedagogical Counselling Centre, 41-902 Bytom, Poland; elizabetkw96@gmail.com

**Keywords:** body dissatisfaction, body image, evaluation of appearance, maternal attitudes toward pregnancy and maternity, pregnancy, prenatal depression

## Abstract

*Background:* Depressive symptoms during pregnancy may cause unfavorable consequences for both the mother and the infant’s physiological and psychological health. Recent evidence indicates that body image plays an important role in prenatal depression. The present study’s main purpose was to investigate the level of acceptance of physical appearance in pregnant women, their attitudes toward pregnancy and maternity, and some obstetric characteristics as significant predictors in the development of depression. *Methods:* A sample of 150 Polish pregnant women completed a set of self-report questionnaires, including the Edinburgh Postnatal Depression Scale (EPDS), Attitudes toward Maternity and Pregnancy Questionnaire (PRE-MAMA), and the Body-Self Questionnaire (EA-BSQ). All participants also answered a brief sociodemographic and obstetric information questionnaire. *Results:* A hierarchical binary logistic regression was conducted to predict prenatal depression from selected obstetric variables (unplanned pregnancy, multiparity, and miscarriages) and psychological variables (appearance evaluation and positive or anxious attitudes toward pregnancy and maternity). It was found that higher levels of negative evaluation of appearance increased chances of depression in pregnant women by almost one-and-a-half. The analysis revealed that positive attitudes toward pregnancy and maternity were the most important protective factor for depression. *Conclusions:* The results confirmed the importance of dissatisfaction with body image during pregnancy as a predictor of the onset of prenatal depression. However, in clinical practice, this risk factor should be considered in combination with positive maternal attitudes, not separately. The implications for future studies and interventions in the field of prenatal depression are discussed in this work.

## 1. Introduction

Perinatal depression is defined as the occurrence of depressive symptoms (major and minor depressive episodes) in women during pregnancy or within the first 12 months after delivery [1,2]. Depression that occurs in the course of pregnancy is known as prenatal depression, while depression that occurs after childbirth defines postnatal or postpartum depression [3]. The prevalence of depression among females is estimated to range from 5% to more than 25% of pregnant women and new mothers [1]. This depends on the recognition criteria [3] or country of residence (women living in low-, middle- and high-income countries—cf. [4]). The prenatal depression experienced by pregnant women has various negative consequences for the wellbeing of the woman, her partner, family, and the birth and development of the child [5]. Furthermore, it increases the risk of postnatal depression [6,7], the consequences of which may be particularly unfavorable for the mother–infant relationship, namely, lower intensity of emotional attachment to the child, adverse effects on the mother–child relationship, delays in cognitive/emotional development of the infant, and behavioral problems in later childhood [3,8,9,10,11,12,13]. Focusing on the consequences of postnatal depression and its risk factors was the subject of many studies [4,5,10,14,15,16,17,18]. 

Not only postpartum depression has a complex etiology. This is also true for prenatal depression, where both obstetric risk factors and social factors are primarily investigated [4]. Among medical and obstetric risk factors, previous miscarriages or stillbirths [4,19,20], unintended or unplanned pregnancies [4,21,22], and multiparity [22] were highlighted. Psychosocial stressors, including low socioeconomic status, unemployment, lower income, fewer years of education, and being a single mother living without a partner [20] should be mentioned. The list of risk factors is much longer and includes stress in the relationship with the partner, maternal anxiety, life stress, prior depression, lack of social support, and domestic violence [4,21].

Another potentially significant risk factor is the negative evaluation of appearance and physical changes during pregnancy. Many women may experience conflicting feelings about body transformation, weight gain, and skin changes [23,24]. Pregnancy is a time of rapid changes specific to each trimester in the body that occur in a relatively short time [25]. Although pregnancy is seen as a special period in a woman’s life where concerns about weight gain and shape may be considered less important, studies showed that many pregnant women still have their standards of appearance formed before pregnancy and feel worried about their physical appearance after delivery [25,26,27]. Across television programs, magazines, movies, and other media, a stereotypical image of femininity is dominated by the thin ideal shape. These social context pressures may contribute to body dissatisfaction and can be a significant predictor of low self-esteem and depression [28]. Scientific evidence suggests that body dissatisfaction in pregnant women is associated with various adverse effects on a woman’s health [29]. Body image dissatisfaction is associated with obesity [25], unhealthy eating behaviors [30], and may play an important role in perinatal depression [23,25,29]. This may consequently have a negative impact on the unborn child [30]. 

Women can see pregnancy as an exceptional time when weight gain is allowed [31], and more eating is accepted [32]. Many pregnant women understand that physical changes are essential for their unborn child’s health and development [27]. Empirical evidence reveals that pregnant women show less dissatisfaction with their body image than nonpregnant women [31,33] and accept more of their actual body size [27]. In most cases, women reported adapting to their bodies’ changes, even though they felt more dissatisfied with their bodies at the beginning and in the middle of the second trimester [29]. Duncombe et al. [34] showed that pregnant women’s body image remains relatively stable throughout pregnancy. Most women can adapt to rapid changes in body size and weight, and attractiveness does not change across pregnancy [34]. Other findings indicate that pregnant women’s perception of the body is generally positive and positively related to self-esteem [35]. However, not all women adapt to changes in the body during pregnancy. Weight gain can be a source of negative body evaluation and a cause for concern that additional unwanted kilograms may be hard to lose after pregnancy [32]. Previous research suggested that most women are increasingly dissatisfied with their appearance as pregnancy progresses [36]. Some women try to control their weight during pregnancy, but others do not do so because they are concerned about possible harm to the baby [26]. 

Psychological adjustment during the transition to motherhood may be easier if pregnant women have positive attitudes toward pregnancy and maternity [37]. This seems to be related to the women’s ability to adapt to multiple changes, including biological, psychological, and social changes [38]. Women who plan a pregnancy have more positive attitudes about pregnancy and are more aware of changes that motherhood may bring [39,40]. There is empirical evidence that positive attitudes toward motherhood and pregnancy represent an important predictor for some aspects of psychological well-being [41], but anxiety attitudes can be negative determinants of the subjective happiness of pregnant women [42]. Maladaptive maternal attitudes predict depressive symptoms during pregnancy and the postpartum period in first-time mothers [43]. Negative attitudes toward pregnancy increase the risk of postpartum depression [43,44]. These two factors are interrelated, although the strength of this relationship is moderate or weak [30,31]. Researchers also found that body image satisfaction is negatively associated with depressive symptoms [23,31] and, according to Fuller-Tyszkiewicz [30], it may be a bidirectional relationship. There are few studies on the predictive effect of body image satisfaction on depressive symptoms during pregnancy. Preliminary results of Rauff and Symons Downs [45] indicated such a prediction, which was also confirmed by other researchers [23].

Although the correlation between body image and depressive symptoms was explored over the last decade, its mechanism remains to be elucidated. Dissatisfaction with body image may be a potential risk factor for depression, whereas a positive attitude to pregnancy may act as a protective factor to mitigate prenatal depression. To our knowledge, empirical studies focusing both on dissatisfaction with body image as a risk factor of prenatal depression in pregnant women and prenatal attitudes toward pregnancy as a potential protective factor have not yet been carried out. In our research, we examine whether these two factors affect the level of depression in pregnant women. As mentioned above, prior research on the relationship between body dissatisfaction and prenatal depression did not result in clear conclusions. Some researchers indicated that most pregnant women are dissatisfied with their body, while others reported positive acceptance of body shape [23]. Therefore, it is important to understand better what factors contribute to prenatal depression in pregnant women and whether satisfaction with physical appearance plays a significant role. 

Based on the presented literature review, the main objectives of this study were as follows: (1) To compare the level of prenatal depression and acceptance of appearance among pregnant women with different obstetric characteristics (parity, planned pregnancy, and miscarriage); (2) to compare the level of prenatal depression and acceptance of appearance among pregnant women with different body image perceptions; and (3) to assess the predictors of the likelihood of depression among pregnant women, such as unplanned pregnancy, primiparous vs. multiparous women, miscarriages, negative/positive attitudes toward pregnancy and motherhood, and acceptance of physical appearance. We hypothesized that women who planned pregnancy and women with no prior experience of miscarriage would have a lower level of prenatal depression and less dissatisfaction with appearance during pregnancy. We also hypothesized that pregnant women who had fewer concerns about body changes caused by pregnancy and were more effective in weight control would have a lower level of prenatal depression and less dissatisfaction with appearance.

## 2. Material and Methods

### 2.1. Participants Characteristics

The sociodemographic characteristics of the sample of 150 pregnant women participating in the study are presented in Table 1. Women were aged between 18–45, mean age was 28 years (*M* = 27.83; *SD* = 4.60). Most of the participants were married (71.8%), the rest were cohabitants (25.5%) or singles (2.7%).

More than half of the participants (64%) were primigravida; for the rest (36%), it was a second or another pregnancy. The group consisted mainly of women with low-risk pregnancies (127 (84.7%)), with only 23 (15.3%) women experiencing high-risk pregnancies. The majority of women completed the questionnaires during the third trimester of pregnancy; the average week of pregnancy was 31 (*M* = 30.95; *SD* = 6.44). More than two-thirds (70%) described their pregnancy as planned, and one fifth had experienced previous miscarriages (20.7%). The full data for obstetric characteristics are shown in Table 2.

### 2.2. Procedure

Data were collected between November 2019 and September 2020 from 150 pregnant women aged 18 or over who were attending routine prenatal check-ups in the obstetric public clinics and obstetric wards at the city hospital in Świętochłowice and in the obstetric clinics of the University Clinical Centre in Gdańsk (Poland). Pregnant women were also recruited in two childbirth education classes in Świętochłowice. All participants completed a set of paper-and-pencil questionnaires voluntarily and anonymously, which were then returned in closed envelopes. 200 sets of questionnaires were distributed and 170 were returned (85%). Twenty questionnaires were rejected due to significant missing data (11.76%). The inclusion criteria were that the women must be Polish-speaking and were not hospitalized on a pregnancy pathology ward. Exclusion criteria: age < 18 years. No incentive was offered for participation in the study. Participants were informed about the purpose of the study and confirmed their consent in writing before answering the questionnaires. The midwives at the specialized public obstetrics clinics and schools of birth collaborated with the researchers and disseminated the information about the study. The study was approved by the Ethics Committee at the University of Silesia in Katowice, Poland (reference no. KEUS.48/05.2020) and was conducted in accordance with institutional ethics standards and the Declaration of Helsinki.

### 2.3. Measures

*Sociodemographic and pregnancy information.* Information on participants’ age, marital status, financial situation, education, residence, and basic pregnancy data was collected using a short sociodemographic questionnaire. 

*The Edinburgh Postnatal Depression Scale* (EPDS) [46]. Maternal, perinatal depression symptoms were measured by the Polish version of the EPDS administered among pregnant women. The EPDS is a 10-item self-report instrument widely used to assess the level of depressive symptoms, mainly after delivery, but also during pregnancy. Respondents answer the items on a 4-point Likert scale, ranging from 0 (absence of symptoms) to 3 (maximum severity of symptoms). A total score is obtained, ranging from 0–30. The acceptable cut-off points for identifying potential perinatal depression range from 9–13, depending on the woman’s culture, language, or personal history of life [47]. It is commonly assumed that cut-off scores ≥10 indicate minor depression and ≥12 indicate major depression [48]. In Jaeschke et al. [49], a score of 13 was adopted as the cut-off point, indicating a high level of depression. In our study, the cut-off point was 12 for identifying potential prenatal depression. The internal consistency of EPDS in our study was α = 0.878.

*The Attitudes toward Motherhood and Pregnancy Questionnaire* (PRE-MAMA) [40] is a self-report instrument to assess attitudes of pregnant women toward pregnancy and the unborn child. The score of the PRE-MAMA may be an important indicator for assessment of the level of adaptation of women to the new period of life (pregnancy and motherhood). The PRE-MAMA scale is an adapted version of the Attitudes to Pregnancy and the Baby scale, which is one of the scales of the multidimensional questionnaire Maternal Adjustment and Maternal Attitude Questionnaire (MAMA) developed by Kumar et al. [37]. The PRE-MAMA consists of 11 items. Each item is rated on a 4-point scale, where 1 means “not at all” and 4 “very much”. This scale encompasses two subscales: Positive Attitude toward Motherhood and Pregnancy (PRE-MAMA1; 6 items, e.g., “Have you been feeling happy that you are pregnant?; “Have you been looking forward to caring for your baby’s needs?”) and Anxious Attitude toward Motherhood and Pregnancy (PRE-MAMA2; 5 items; e.g., “Have you been worrying that you might not be a good mother”; “Have you felt that life will be more difficult after the baby is born?”). In our study, the internal consistency for PRE-MAMA1 was α = 0.711 and α = 0.685 for PRE-MAMA2. 

*The Body-Self Questionnaire* (BSQ) developed by Sakson-Obada [50,51] is a self-report instrument to assess different body-self aspects. We used one of the subscales, namely the Appearance Evaluation (EA-BSQ), to assess the affective aspects of body image, particularly satisfaction with appearance. This subscale consists of 8 items related to the evaluation of body image (e.g., “I feel physically attractive”; “I am ashamed of my appearance.”; “The appearance of some parts of my body evokes negative feelings in me such as anger, shame, loathing, etc.”). Each item is answered on a 5-point scale (1 = “not true at all”; 5 = “very true”). Higher scores indicate a higher level of negative evaluation of body image and dissatisfaction with appearance. In our sample, the internal consistency was α = 0.863.

### 2.4. Statistical Analysis

The study analyses were conducted using the Statistical Package for Social Sciences version 22 (IBM SPSS Statistics 22; SPSS Inc., Chicago, IL, USA). Descriptive statistics were computed for sociodemographic characteristics. Further, to examine possible differences in the level of prenatal depression and the evaluation of appearance (satisfaction with appearance) among pregnant women with different obstetric factors and body image perceptions, we used the Mann–Whitney U-test or the Kruskal–Wallis test for group comparisons (the analyzed groups differed in size). Pearson’s rank correlation analysis was used to assess correlations between symptoms of prenatal depression and other psychological variables. Hierarchical binary logistic regression analysis was performed to describe factors associated with the likelihood of depression. The value of *p* < 0.05 was assumed for the significance level. To determine the probability of detecting an effect of a given size with a given level of confidence under constraints of the sample size, we also conducted a post-hoc power analysis in GPower 3.1.9.4 (University of Düsseldorf, Germany). This analysis suggested that the sample size provided sufficient statistical power for each statistically significant predictor (>0.90).

## 3. Results

### 3.1. Comparative Analysis of Symptoms of Depression and Acceptance of Appearance in Pregnant Women with Different Obstetric Characteristics and Perception of Body Image Changes

A high level of specific concerns about body changes caused by pregnancy was reported by one-fifth of women (18%); the rest (82%) reported moderate or small intensity of such concerns. However, the percentage of pregnant women who did not accept or only partly accepted their current physical appearance was higher (36%) than those who declared a high level of specific concerns about body changes caused by pregnancy. Total acceptance of the current appearance was reported by 64%. Moreover, most women were convinced of low effectiveness of weight control (fully effective = 30%; partly or ineffective = 70%). All these data are shown in Table 2. The comparative analyses presented in Table 2 indicated significant differences between women with a previous history of pregnancy losses and those without miscarriages. The women with miscarriages experienced more depressive symptoms during pregnancy and more negative appearance evaluation. The analysis did not reveal statistically significant differences in depression symptoms and negative appearance evaluation in terms of parity (primipara vs. multipara) and pregnancy planning (planned vs. unplanned pregnancy). Body image perception did vary in women with a higher intensity of prenatal depression and negative physical appearance evaluation. Women who did not accept or only partly accepted their current physical appearance showed a higher level of intensity of prenatal depression and scored higher on the EA-BSQ scale. Furthermore, women who were convinced of the low effectiveness of weight control reported higher intensity of prenatal depression. Finally, women who were very strongly concerned about physical changes in their bodies showed more negative appearance evaluation.

### 3.2. Satisfaction with Appearance and Prenatal Attitudes toward Pregnancy and Maternity as Predictors of Depression

Table 3 displays the median and intercorrelations among the main study variables for the total sample. The symptoms of depression positively correlated with negative evaluation of appearance and anxious attitude toward pregnancy, and negatively correlated with positive attitude toward pregnancy and motherhood. The negative evaluation of appearance was positively correlated with the age of pregnant women and anxious pregnancy attitudes, and was also negatively correlated with positive pregnancy attitudes.

The study showed that 22% of pregnant women belonged to the group with symptoms, probably indicating a clinical level of depression. According to the standard EPDS cut-off scores, 33 out of 150 pregnant women screened positive for depression. These women obtained a score of 12 points or above.

Hierarchical binary logistic regression analyses were carried out to calculate the adjusted odds ratio (AOR) for women who scored 12 or more and were probably experiencing a higher intensity of depressive symptomatology. As shown in Table 4, a hierarchical binary logistic regression was conducted to predict depression from selected obstetric variables (unplanned pregnancy, multiparity, miscarriages) and psychological variables (evaluation of appearance, positive or anxious attitudes towards pregnancy and maternity). The first step of the model covered obstetric characteristics and predicted 6% of the variance in depressive symptoms, with unplanned pregnancy (AOR = 2.84, *p* = 0.013) increasing the risk of severe symptoms of prenatal depression. In the final model (total *R*^2^ = 0.59), positive attitudes toward pregnancy and maternity (AOR = 0.78, *p* = 0.013) and negative evaluation of appearance (AOR = 1.42, *p* = 0.001) independently predicted a greater likelihood of prenatal depression. 

The high levels of negative evaluation of appearance increased the chances of depression likelihood in pregnant women by almost one-and-a-half (AOR = 1.42). Analysis also revealed that a positive attitude was an important protective factor for depression (AOR = 0.78). 

## 4. Discussion

This study investigated how dissatisfaction with physical appearance and maternal attitudes during pregnancy are associated with prenatal depression. In particular, it was sought whether a negative assessment of appearance combined with anxious attitude to pregnancy and some obstetric characteristics could be risk factors for prenatal depression and whether a positive maternal attitude could have a mitigating influence on depression in pregnant women. Further, we compared the level of prenatal depression and acceptance of appearance among pregnant women differing in (1) selected obstetric characteristics (parity, planned pregnancy, and miscarriage) and (2) body image perception.

The regression analysis results confirmed the importance of dissatisfaction with the body during pregnancy as a significant predictor of the onset of prenatal depression. Interestingly, the negative assessment of physical appearance increased the chances of depression by almost one-and-a-half. These findings are in line with previous studies showing that body image dissatisfaction is an important factor predicting depression in women during pregnancy [23,45]. Other researchers also pointed out that pregnant women feeling more body concerns experience higher depressive symptoms [34]. It is possible that those women reported more worries about body shape and size because they anticipated more difficulties to cope with it after delivery [25,26]. 

Another important finding of the regression analysis concerned positive attitudes toward pregnancy and motherhood. It turned out that this variable is a significant protective factor for prenatal depression. This means that specific beliefs about motherhood can facilitate the process of women’s adaptation to multiple changes in life, including those that occur in their bodies. A similar conclusion can be drawn from Skouteris et al. [29], i.e., that most women adapt to bodily changes. It is probably easier for them to explain to themselves that pregnancy is a special time in a woman’s life, when concerns for physical appearance standards are not as important as the child’s health [27,31]. Positive attitudes protect the wellbeing of the mothers and support adaptation during pregnancy [40,41]. Pregnant women who enjoy and look forward to motherhood’s challenges are more aware of the need for changes, and accept them [39].

The obtained results suggested that risk factors for antenatal depression should be considered together with protective factors. In our study of depression predictors, we combined variables that could potentially be risk factors, such as selected pregnancy characteristics (parity, unplanned pregnancy, and miscarriages) and psychological risk determinants (negative evaluation of appearance and anxious attitude toward pregnancy and maternity) with protective factors (positive attitudes toward pregnancy and maternity). In contrast with the previous findings of other researchers [4,21,22], the obstetric variables, for instance, multiparity, miscarriages, and unplanned pregnancy, were not statistically significant predictors of depression in the final model of regression analysis. Two predictors were important, namely, negative evaluation of appearance (risk factor) and positive maternal attitude (protective factor).

The idea of combining positive and negative factors as determinants of mental health was undertaken in studies on adaptation to pregnancy [41,42,52,53,54]. A similar approach also appeared in the study by Hain et al. [55] on antepartum and postpartum depression. Our research is in line with that trend.

Interestingly, we also revealed some inconsistency in pregnant women’s self-perception (most of them were in the third trimester). Women who were convinced of the low effectiveness of weight control (70%, cf. Table 2) reported higher intensity of prenatal depression. On the other hand, 64% of the participants declared full acceptance of their current appearance. A possible explanation for this finding is that women mostly adapt well to body changes, even if they feel unsatisfied [29]. Pregnant women accept more their body size and make fewer attempts to control their weight [27]. Many women are convinced that pregnancy legitimizes their weight, allows them not to worry about it, and diminishes the pressure to be slim [27,31]. Thus, greater differences in the deviation from the ideal body shape do not necessarily result in a higher level of dissatisfaction with the body [27]. Women modify “their self-standards for their body over the course of pregnancy” [34]. However, for some women, gaining weight is associated with feeling discomfort due to social pressure to be slim [28]. For those women, pregnancy increases body dissatisfaction and depressive symptoms [31]. Women with great body concerns before pregnancy are likely to maintain body concerns during pregnancy and feel depressive symptoms [34].

As we can see, clear conclusions about the impact of pregnancy on women’s satisfaction with their bodies are difficult to draw [33]. Understanding psychological adaptation during pregnancy requires consideration of many factors that may affect prenatal adaptation [52]. Researchers and practitioners often consider such factors as obstetric features, marital status, social and partner support, personality traits, antenatal depression, and physical or mental health problems. Our findings suggest that health professionals, midwives, and psychologists working with pregnant women should also consider the negative evaluation of appearance and body dissatisfaction as a risk factor and positive maternal attitudes toward pregnancy and motherhood as a protective factor. We agree with the conclusion of Guzman-Ortiz et al. [56] that an important task for prenatal care professionals is to consider assessing body image during pregnancy. This would allow pregnant women (especially in the middle of their second or early third trimester) with body image problems to be identified, help them accept changes in their physical appearance, and protect them from perinatal depression. Although such serious dissatisfaction with body image is experienced by the minority of women, it may have major consequences for their mental health and the health of the unborn babies. Besides depression, dissatisfaction with body image can cause unhealthy eating behaviors during pregnancy. Our study suggests the need to include the risk of body dissatisfaction in antenatal education programs of childbirth classes.

The limitations of the current study are related to the representativeness of the sample. The investigation was cross-sectional and nonrandom sampling was used to select pregnant women. Moreover, almost all of the participants were married or cohabiting with partners. Our sample was rather homogeneous concerning education and socioeconomic status. The majority of participants were highly educated, and only a few women had elementary education. Therefore, the generalizability of the findings to a more diverse population of pregnant women is limited. Additional research is needed to replicate these findings on sociodemographically diverse samples of pregnant women. In particular, future studies should rely on a longitudinal design and make measures in two or more points of time across pregnancy (e.g., comparison of second and third trimesters). Moreover, Body Mass Index (BMI) should also be monitored in pregnant women. This objective index may be a potentially important factor differentiating women in terms of body acceptance. It would also be worth investigating the body image acceptance among women in the same week of pregnancy. Despite these limitations, we believe this study contributes to the knowledge on dissatisfaction with appearance and prenatal maternity attitudes as determinants of antenatal depression in pregnant women. 

In conclusion, our findings supported the importance of dissatisfaction with body image during pregnancy as an important predictor of the onset of prenatal depression. The negative evaluation of appearance predicted a greater likelihood of depressive symptoms. Furthermore, positive attitudes toward pregnancy and motherhood represent a significant protective factor for depression in pregnant women. Thus, it is suggested that both factors should be considered jointly (not separately) in clinical practice when assessing the risk of developing prenatal depression in pregnant women. 

## Figures and Tables

**Table 1:** Sociodemographic characteristics of pregnant women (*N* = 150).

Sociodemographic Variable	Mean (*SD*)	*n* (%)
**Age**	27.83 (4.60)	
**Marital status**		
Married		107 (71.8)
Cohabitants		38 (25.5)
Single		4 (2.7)
**Place of residence**		
Village		13 (8.7)
Small city (<50,000 citizens)		28 (18.7)
Medium city (50,000–100,000 citizens)		41 (27.3)
Large city (>100,000 citizens)		68 (45.3)
**Education**		
Elementary education		8 (5.3)
Secondary education		51 (34.0)
Higher education		91 (60.7)
**Financial status**		
Bad or average		63 (42.0)
Good or very good		87 (58.0)

Note: *SD*—standard deviation.

**Table 2:** Mean ranks of prenatal depression (EPSD) and the evaluation of appearance (EA-BSQ) in pregnant women with different obstetric factors and body image perception (*N* = 150).

Category	*n* (%)	EPSD Mean Rank	EA-BSQ Mean Rank
**Parity**		*Z* = 1.14	*Z* = 1.83
Primipara	96 (64.0)	72.46	70.65
Multipara	54 (36.0)	80.91	84.13
**Planned pregnancy**		*Z* = −1.53	*Z* = −0.313
Yes	105 (70.0)	71.96	74.78
No	45 (30.0)	83.77	77.19
**Previous pregnancy losses**		*Z* = 1.07 *	*Z* = 2.66 **
Yes	31 (20.7)	92.73	93.95
No	119 (79.3)	71.01	70.69
**Acceptance of the current appearance**		*Z* = −5.35 ***	*Z* = −8.09 **
No or partly acceptance	54 (36.0)	100.75	113.69
Fully acceptance	96 (64.0)	61.30	54.02
**Concerns about body changes caused by pregnancy**		*Z* = 1.87	*Z* = 3.42 ***
Small or moderate	123 (82.0)	72.39	69.83
Very strong	27 (18.0)	89.67	101.35
**Effectiveness of weight control**		*Z* = 2.63 **	*Z* = 1.57
Yes, fully effective	45 (30.0)	61.27	67.01
Partial or ineffective	105 (70.0)	81.60	79.14

*Note*: EPSD—symptoms of depression; EA-BSQ—evaluation of appearance; *** *p* < 0.001; ** *p* < 0.01; * *p* < 0.05.

**Table 3:** Median, reliabilities, and intercorrelations between the study variables.

Measure	*α*	*Me*	EPSD	EA-BSQ	PRE-MAMA1	PRE-MAMA2	Age	Week of Pregnancy
EPSD	0.878	8.00	1					
EA-BSQ	0.863	16.00	0.629 **	1				
PRE-MAMA1	0.711	21.00	−0.322 **	−0.271 **	1			
PRE-MAMA2	0.685	11.00	0.325 **	0.309 **	−0.024	1		
Age		27.83	0.039	0.200 *	0.007	0.087	1	
Week of pregnancy		30.95	0.070	−0.120	−0.047	−0.037	−0.150	1

*Note:* EPSD—symptoms of depression; EA-BSQ—Evaluation of Appearance; PRE-MAMA1—Positive Attitude toward Pregnancy and Motherhood; PRE-MAMA2—Anxious Attitude toward Pregnancy and Motherhood; ** *p* < 0.01; * *p* < 0.05.

**Table 4:** Hierarchical binary logistic regression predicting depression (*N* = 150).

Predictors	Step 1		Step 2	
	AOR	95% CI	AOR	95% CI
Unplanned pregnancy	2.84 *	1.24–6.49	3.48	0.92–13.04
Multiparity	1.14	0.49–2.63	0.72	0.19–2.67
Miscarriages	1.01	0.35–2.94	0.50	0.11–2.14
PRE-MAMA 1 PRE-MAMA 2			0.78 * 1.18	0.64–0.94 0.98–1.42
EA-BSQ			1.42 ***	1.23–1.63
	*R*^2^ = 0.06 *		*R*^2^ = 0.59 ***	

*Note:* AOR—Adjusted Odds Ratio; CI—Confidence Interval; EA-BSQ—Evaluation of Appearance; PRE-MAMA1—Positive Attitude toward Pregnancy and Motherhood; PRE-MAMA2—Anxious Attitude toward Pregnancy and Motherhood; * *p* < 0.05; *** *p* < 0.001.
